# Concentration of propolis as a storage medium for avulsed teeth: a systematic review

**DOI:** 10.3389/fmed.2025.1583113

**Published:** 2025-10-02

**Authors:** Chaya Chhabra, Kumar Gaurav Chhabra, Seemadevi Thangeswaran, Shraddha Shere

**Affiliations:** ^1^Department of Paediatric and Preventive Dentistry, Nims Dental College & Hospital, Nims University Rajasthan, Jaipur, India; ^2^Department of Public Health Dentistry, Nims Dental College & Hospital, Nims University Rajasthan, Jaipur, India; ^3^Adjunct Faculty, Research & Development Cell, Datta Meghe Institute of Higher Education & Research, Wardha, Maharashtra, India

**Keywords:** avulsed tooth, avulsion, storage medium, propolis, PDL cells

## Abstract

**Introduction:**

Dental avulsion is among the most serious types of traumatic tooth injuries, involving the total displacement of the tooth from its socket within the alveolar bone. This form of injury causes detrimental effects to surrounding structures, including the periodontal ligament (PDL) & bone, cementum. Immediate transplantation of the avulsed tooth is highly advocated, as it plays a critical role in determining the success and prognosis of treatment. The primary objective of this systematic review was to assess the effectiveness of various concentrations of propolis as a storage medium for avulsed teeth, focusing on the survival capacity of PDL cells.

**Methods:**

This literature review was carried out in accordance with the PRISMA guidelines, ensuring a transparent and systematic approach to study selection and reporting. Articles were sourced from multiple reputable databases, including Cochrane, PubMed, ScienceDirect, Scopus, Web of Science, and Google Scholar, to comprehensively identify relevant studies for inclusion. The articles were reviewed for initial reading using ZOTERO software. The methodology of the selected research studies was then assessed using the QUIN tool, which is designed to assess the quality of *in vitro* studies.

**Results:**

The initial search identified 30 articles, of which 21 underwent full-text review. Based on the predefined eligibility criteria, 11 research articles were ultimately chosen for qualitative analysis. The findings revealed that at 30 min of extraoral dry time, propolis preserved the highest number of viable periodontal ligament (PDL) cells compared to other storage media, such as HBSS, milk, coconut water, and pomegranate juice. Propolis has been studied at various concentrations, with 10% propolis showing the most promising results. It not only ensured a high number of viable PDL cells but also preserved PDL cell viability over extended periods, including 3, 6, 12, and 24 h.

**Conclusion:**

10% Propolis demonstrated superior effectiveness in preserving PDL cell viability compared to other storage media. Based on these findings, propolis can be considered a preferable alternative for preserving the sustainability of PDL cells in avulsed teeth.

## Background

Trauma to the oral and maxillofacial region is a common occurrence accounting for approximately 6% of all classified injuries for which individuals need to Strive for treatment (1). Among these, traumatic dental injuries were particularly frequent with crown fractures and luxation being the most commonly observed types. Tooth avulsion, a tooth completely displaced from its alveolar socket is especially prevalent in permanent anterior teeth during childhood affecting 0.6 to 16% of children aged 7 to 10 years (2). Avulsion typically occurs as a result of everyday activities or sporting events, including falls, accidents, or injuries during physical activities. Sports contribute to 60% of traumatic dental injuries (TDI) with schools being a primary location for these incidents surpassing other environments (3). According to the World Health Organization (WHO), exarticulation or tooth avulsion, is described as the complete displacement of a tooth from its alveolar socket as a result of traumatic injury (4). Immediate replantation of an avulsed tooth is crucial for preserving the viability of the periodontal ligament (PDL) cells which are essential for the prolonged or sustained survival and prognosis of the relocated tooth (5). The periodontal ligament (PDL) plays a vital role in the healing process of replanted teeth and preserving its vitality is key to ensuring successful reattachment. Immediate replantation of the tooth is not always feasible due to various factors, such as the location of the injury, availability of professional care, or the patient’s condition. In such cases, the avulsed tooth should be stored in an appropriate medium to prevent dehydration, maintain cell viability and protect the periodontal ligament. Ideal storage media include milk, saline, or specialized solutions like Hank’s Balanced Salt Solution, as they provide the necessary nutrients and osmolarity to support cell survival until replantation is possible. Prompt and proper handling of the tooth is critical to improve the chances of successful reattachment and long-term prognosis.

Two critical factors that influence the success of replantation are the extraoral dry time (the time the tooth remains outside the socket) (The duration the tooth stays outside the socket) and the storage medium used. Although both factors are important, the capacity of a storage medium to maintain the viability of periodontal ligament (PDL) cells is considered more crucial in preventing complications like ankylosis and replacement resorption (6). Numerous varieties of storage media, which is wet in consistency have been explored for avulsed & also been investigated (4).

However, as science evolves and new materials are discovered, there is a growing need for innovative storage media that can offer better results. Propolis, a natural resin collected by bees from plant buds, has recently emerged as a promising alternative for preserving avulsed teeth. Known for its antifungal, antibacterial, and anti-inflammatory properties. Propolis has shown potential in maintaining PDL cell viability surpassing traditional media like HBSS in some studies (5). Propolis is composed of a variety of substances including 50% of resin and vegetable balsam, 30% of wax, 10% of essential oils, and 5% of pollen among others. Its composition can vary significantly depending on environmental factors making it a dynamic material. Given its potential benefits propolis may offer a new and effective alternative to traditional storage media. As scientific research continues to advance it is essential to explore novel storage media for avulsed teeth that can further improve the preservation of Periodontal Ligament cell viability and enhance the chances of successful tooth replantation. This systematic review seeks to evaluate the effectiveness of different concentrations of propolis as a storage medium providing significant insights into its potential as a contemporary solution for managing tooth avulsion.

## Methods

The protocol for this systematic review was registered in the International Prospective Register of Systematic Reviews (PROSPERO) (7) under the reference number CRD42024576620. This review is centered on *in vitro* studies (laboratory-based studies or *test-tube experiments*) lack information derived from patients, as *in vivo* studies have not been conducted till now. The PICOS framework [Population (P): Human PDL cells isolated from freshly extracted teeth, Intervention (I): Propolis, Comparison (C): Control and other storage media (e.g., HBSS, milk, coconut water, etc.), Outcome (O): PDL cell viability & Study Design (S): *In vitro* studies] for the research question was as follows: “Is propolis a more effective storage medium than other media in maintaining cell viability in avulsed teeth?”

Exclusion criteria included studies involving fractured teeth, periodontal disease, or carious teeth, as well as animal-based studies, ongoing trials, case reports/series, narrative reviews, short communications, letters to editors, cross-sectional studies, and non-English publications. This systematic review adheres to the AMSTAR-2 (8) (A Measurement Tool to Assess Systematic Reviews) guidelines.

The objective of the search method as to locate research studies that examine the impact of propolis as a preservative medium in Preserving the life span or survivability of PDL cells from avulsed teeth in laboratory conditions. Three authors conducted the comprehensive literature review and search method autonomously. The systematic literature search was carried out across multiple databases, including PubMed/MEDLINE, Cochrane, ScienceDirect, Scopus, Web of Science, Google Scholar, and EMBASE, from inception to December 2024.

The search process was designed using the mentioned MeSH (Medical Subject Headings) keywords: [propolis, tooth avulsion]. These terms were strategically chosen to ensure a thorough and targeted retrieval of relevant studies, aligning with the objectives of the review and maximizing the scope of the literature search, they were: “Propolis for avulsed teeth OR Dental Trauma teeth,” “Honey bee extract OR Propolis for avulsed teeth” “Knocked out teeth OR Knocked out tooth OR chipped out teeth,” “Tooth avulsion OR Dental Avulsion,” “Cell OR Periodontal Ligament Cell survival of avulsed tooth,” “Cell viability of avulsed tooth OR PDL Cell Viability,” “Tooth replantation OR implantation,” and “Periodontal ligament in avulsed teeth OR Avulsed teeth.” The “AND” and “OR” Boolean operators were applied to combine keywords: [“propolis for avulsed teeth”*] OR biological transport of avulsed tooth, [“cell survival of avulsed tooth”*] OR cell viability of avulsed tooth, [“tooth replantation”*] AND [“periodontal ligament in avulsed teeth”*] AND [“Honey bee extract for avulsed teeth OR knocked out teeth*”]. The literature search was conducted in alignment with the predefined inclusion criteria to ensure the selection of studies relevant to the research objectives.

**Figure 1:**
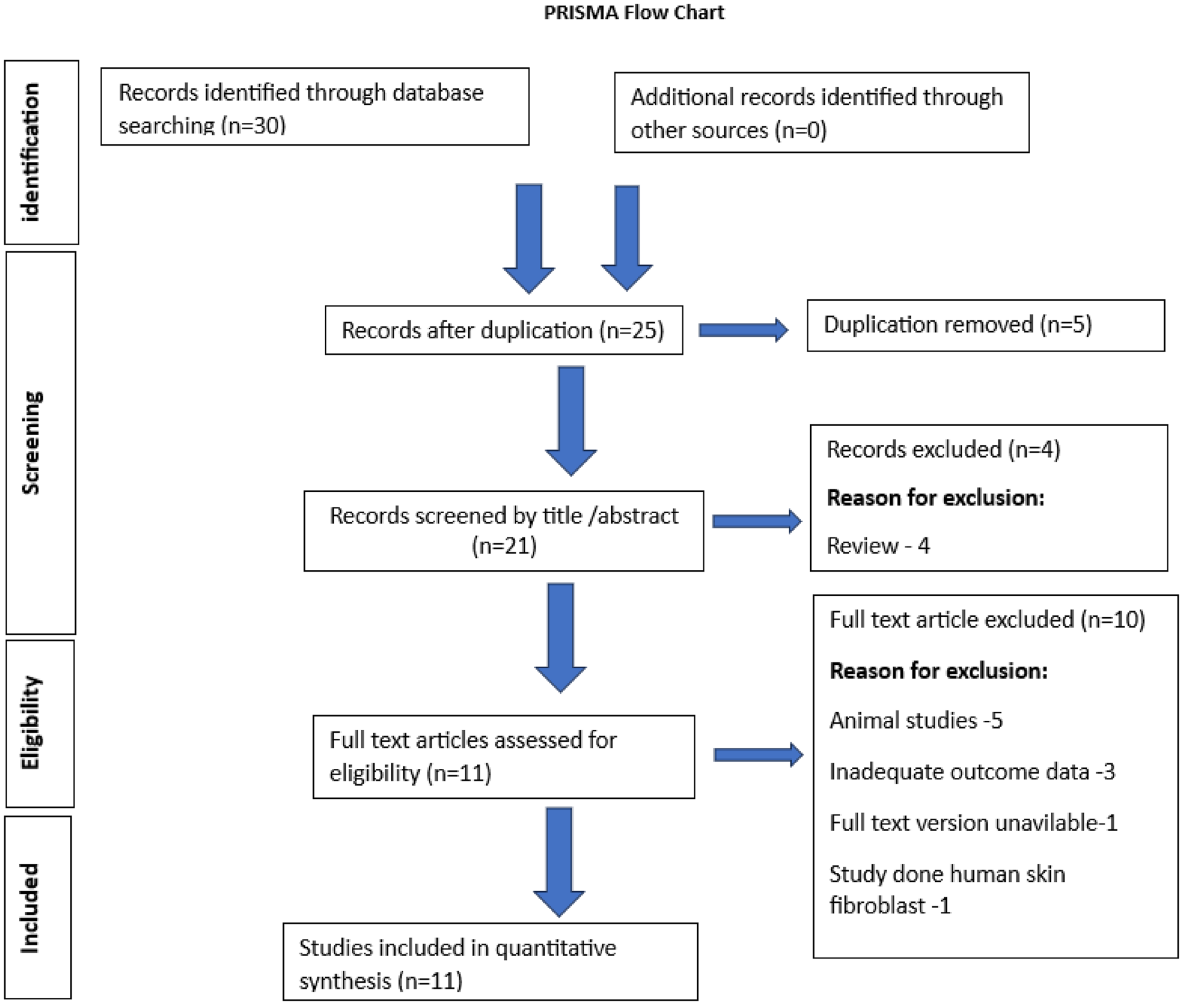
PRISMA flowchart of the study.

The study selection process for this review is illustrated in Figure 1. Initially, all duplicate references were removed using Zotero reference management software (9) to ensure a streamlined review. The selection of the study was carried out in dual phases.

In the primary phase, three authors independently reviewed the titles and abstracts of all the identified studies to assess their relevance based on predefined inclusion criteria. Studies that appeared relevant at this stage were moved to the second phase for full-text review.

During the second phase, the same three authors and additional 2 authors thoroughly read the full texts of the remaining studies to evaluate their eligibility for inclusion in the systematic review. At this stage, all relevant data from the studies, such as study design, sample characteristics, outcomes, and methodologies, were collected and carefully recorded.

To ensure accuracy and reduce bias, a third and fourth author independently checked the extracted information. Finally, key details from all the included studies were summarized in Table 1, highlighting important findings and methodological aspects.

**Table 1:** Summary of studies included in our review.

Author & year	Study design	Participant/research target	Intervention	Comparator	Method of assessment	Outcome (% of viable cells in different time periods)
Martin MP et al. (11) 2004	*In vitro*	Seventy freshly extracted human teeth	Saline (*n* = 12) Milk (*n* = 12) HBSS (*n* = 12) 50% propolis (*n* = 10) 100% propolis (*n* = 12)	Positive control (*n* = 5) – not stored in any storage medium nor dried Negative control (*n* = 5) -bench dried for 8 h	0.4% trypan blue	The teeth stored in 50% propolis and 100% propolis demonstrated highest number of PDL viable cells than other storage media at 30 min, there is no significant difference between 50% propolis and 100% propolis
Ozon F et al. (12) 2007	*In vitro*	Freshly Extracted 3rd molar teeth	10% propolis 20% propolis, Milk HBSS	DMEM- positive control Tap water Negative control	0.4% Trypan blue	10% propolis was a more effective storage medium then other groups & it showed highest % of cell viability at 1,3,6,12,24 h.
Gopikrishna V et al. (13) 2008	*In vitro*	Seventy freshly extracted human teeth	Coconut water (*n* = 15) Propolis (*n* = 15) HBSS (*n* = 15) Milk (*n* = 15)	Positive control (*n* = 5) – 0 min dry time Negative control (*n* = 5) – 8 h dry time	0.4% trypan blue	Coconut water demonstrated highest number of viable PDL cells than propolis and other storage medium.
Saxena P et al. (14) 2011	*In vitro*	Extracted teeth	Propolis 2.5% Propolis 5% Propolis 10% Propolis 20% HBSS Milk (0.5%) Artificial saliva DMEM Propolis10% + DMEM Propolis20% + DMEM	–	0.4% trypan blue	Combinations of propolis 10% + DMEM, propolis 20% + DMEM, and DMEM alone were found to be better than other media used in this study at 30 min, 1,3,6,12,24 h
Ahangari Zohren et al. (15) 2012	*In vitro*	60 freshly extracted anterior single root teeth	propolis 10% (*n* = 10) propolis 50% (*n* = 10) HBBS (*n* = 10) Milk (*n* = 10) Egg white (*n* = 10)	Positive control Control (*n* = 5) – immediately after extraction Negative control (*n* = 5) – dried for 12 h	0.4% trypan blue	Propolis showed significantly more viable PDL cells than other storage medium at 1 h. At 3 h, 10% propolis showed more viable cells than 50% propolis followed by HBSS, egg white and milk.
Najeh Saana et al. (5) 2013	*In vitro*	Sound permanent first molars extracted for orthodontic purpose	DMEM Ethanolic propolis solution Propylene glycol propolis solution Mature coconut water	Untreated cells	MTT assay	soaking in mature coconut water only resulted in higher percentages of viable cells at 0,30,45, ≥60 min
Babaji Prashant et al. (16) 2017	*In vitro*	Fifty extracted premolar teeth	HBSS (*n* = 10) 50% propolis (*n* = 10) *Aloe vera* (*n* = 10) pomegranate (*n* = 10)	Positive control (*n* = 5) – treated with collagenase dispase Grade II negative control (*n* = 5)- bench drying for 8 h	0.4% trypan blue	Viability of cells in decreasing order is positive control > propolis > HBSS > *A. vera* > PJ > negative control. 50% propolis showed high viable cells at 45 min.
XJ Yuvan et al. (17) 2018	*In vitro*	Extracted premolars & wisdom teeth	Brazilian propolis (BP) HBSS Milk Tap water	–	Cell Counting Kit (CCK-8) assay	Cell viability was the highest in the milk group followed by the BP and HBSS groups. Most cells died when incubated in tap water
Shingare Poonam et al. (18) 2020	*In vitro*	50 freshly extracted premolar teeth	Propolis (*n* = 10) Milk (*n* = 10) Egg albumin (*n* = 10)	Positive Control (*n* = 10) -immediately assessed after extraction Negative control (*n* = 10) – bench dried for 8 h	0.5% trypan blue	Propolis demonstrated highest number of viable PDL cells followed by milk and egg albumin at 30 min.
Misurya R et al. (19) 2022	*In vitro*	40 freshly extracted teeth	ViaSpan (*n* = 10) *Aloe vera* (*n* = 10) Gatorade (*n* = 10) Propolis (*n* = 10)	–	0.4% trypan blue	Highest number of viable PDL cells observed in propolis group followed by Viaspan, *Aloe vera* and Gatorade solution at 3,6,24,48,72 h.
Thoyalil musaffar et al. (6) 2023	*In vitro*	Sixty freshly extracted premolars	Placentrex (*n* = 15) propolis 10% (*n* = 15) pomegranate juice 5% (*n* = 15) HBSS (*n* = 15)	–	0.4% trypan blue	HBSS showed highest number of viable PDL cells than other storage medium. Followed by Placentrex showed significantly more viable PDL cells than pomegranate and propolis at 30 min

The risk of bias (RoB) assessment was performed by the same two authors and subsequently reviewed by an expert using the QUIN tool (10) (Quality Assessment Tool for *In Vitro* Studies). The following aspects were evaluated for ROB: clearly stated aims/objectives, sample size calculation, comparator group details, methodology explanation, randomization, outcome measurement methods, statistical analysis, and presentation of results.

Each parameter was assessed and assigned a score based on its level of specification: “not specified” (score = 0), “inadequately specified” (score = 1), or “adequately specified” (score = 2). The overall Risk of Bias (ROB) percentage was then calculated using the following equation:


ROB(%)=(total score×100)/(2×applicable criteria)

This formula ensures a standardized and proportional evaluation of the ROB across studies, taking into account the number of applicable criteria.

Based on their ROB scores, the studies were categorized into the following risk levels:

- **High risk:** ROB score < 50%.
- **Medium risk:** ROB score between 50 and 70%.
- **Low risk:** ROB score >70%.

This classification provided a clear framework for evaluating the methodological quality and reliability of the included studies.

## Result

A total of 30 records were identified through database searching. After the removal of five duplicate records, 25 articles remained. These were screened by title and abstract, resulting in the exclusion of four articles, all of which were review papers. The remaining 21 articles were assessed for full-text eligibility. Of these, ten were excluded for various reasons: five were animal studies, three did not provide adequate outcome data, one lacked an accessible full-text version, and one study was conducted on human skin fibroblasts rather than relevant dental tissues. Following this screening process, eleven studies met the inclusion criteria and were included in the quantitative synthesis (5, 6, 10–19). The entire process is outlined in a PRISMA flow chart diagram (Figure 1).

The characteristics of the included studies of our review were analyzed. All articles focused on *in vitro* studies examining the effects of propolis on avulsed periodontal ligament (PDL) tissues in humans. However, each study utilized different methods to evaluate these effects, as detailed in Table 1.

Of the 11 studies investigating the impact of propolis on avulsed teeth, 9 reported positive effects on PDL cells. Additionally, all studies assessed the primary cultures of cells & treating PDL culture with different storage media at different time points or intervals, with no research examining cells from other dental tissues such as cementum, gingiva, or alveolar bone.

Cell viability was evaluated in all 11 studies using various methods. Trypan blue (0.4%) was the most frequently used, appearing in 10 studies, while one study employed a 0.5% concentration. The MTT assay was used in one study, and another utilized the Cell Counting Kit-8 (CCK-8) assay to assess cell viability.

All studies included in our review utilized a standard comparator, such as HBSS, coconut water, milk, artificial saliva, egg white, *aloe vera*, pomegranate, Placentrex, and Gatorade. In 9 out of the 11 studies, propolis demonstrated excellent results in maintaining cell viability. Additionally, comparisons were made across various propolis concentrations (10, 20, 50, and 100%), with 10% propolis showing the highest mean of viable cells, followed by the other concentrations in descending order (10 < 20 < 50 < 100%).

Within the first 30 min of extra-oral dry time, propolis demonstrated the highest number of viable cells when compared to other storage media. This trend continued over longer periods of extra-oral dry time (3, 6, 12, and 24 h), where propolis consistently maintained better cell viability. This indicates that propolis is effective in both short-term and long-term storage scenarios for avulsed teeth.

However, in 2 out of the 11 studies, coconut water outperformed propolis in terms of cell viability. These studies employed different methods to evaluate cell viability, specifically using the MTT assay, which may account for the variations in results. This discrepancy suggests that the method used to assess cell viability might influence the outcomes, leading to conflicting results in the comparison between propolis and coconut water.

Additionally, one study compared propolis with Placentrex, concluding that Placentrex exhibited a higher percentage of viable cells than propolis. This suggests that Placentrex may offer an alternative and possibly more effective solution for maintaining cell viability, though further research is necessary to validate these findings.

Since the included studies were *in vitro*, many parameters typically used in the risk assessment of clinical trials—such as blinding, sampling techniques, operator details, and outcome assessor details were not applicable. Therefore, these factors were not considered for the risk assessment in our review.

The risk of bias (ROB) for each study was evaluated using the QUIN (Quality Assessment Tool for *In Vitro* Studies) (10). This tool assesses bias across 8 key parameters: clearly stated aims/objectives, sample size calculation, group details, methodology explanation, randomization, methods of outcome measurement, statistical analysis, and the presentation of results (Table 2). All the studies demonstrated a low overall risk of bias.

**Table 2:** Detailed information regarding the ROB analysis.

Article	C1	C2	C3	C4	C5	C6	C7	C8	Final score	% of ROB	Overall ROB
Martin MP et al. (11) 2004	2	0	2	2	1	2	2	2	13	81%	Low risk
Ozon F et al. (12) 2007	2	0	2	2	0	2	2	2	12	75%	Low risk
Gopikrishna V et al. (13) 2008	2	0	2	2	1	2	2	2	13	81%	Low risk
Saxena P et al. (14) 2011	2	0	2	2	0	2	2	2	12	75%	Low risk
Ahangari Zohren et al. (15) 2012	2	0	2	2	1	2	2	2	13	81%	Low risk
Najeh Saana et al. (5) 2013	2	0	2	2	0	2	2	2	12	75%	Low risk
Babaji Prashant et al. (16) 2017	2	0	2	2	1	2	2	2	13	81%	Low risk
XJ Yuvan et al. (17) 2018	2	0	2	2	0	2	2	2	12	75%	Low risk
Shingare Poonam et al. (18) 2020	2	0	2	2	1	2	2	2	13	81%	Low risk
Misurya R et al. (19) 2022	2	2	2	2	1	2	2	2	15	93%	Low risk
Thoyalil musaffar et al. (6) 2023	2	0	2	2	1	2	2	2	13	81%	Low risk

However, only one study included a sample size calculation, which resulted in the highest % of RoB score at 93%. Moreover, 4 studies did not employ randomization, contributing to a 75% RoB for those particular studies.

**Figure 2:**
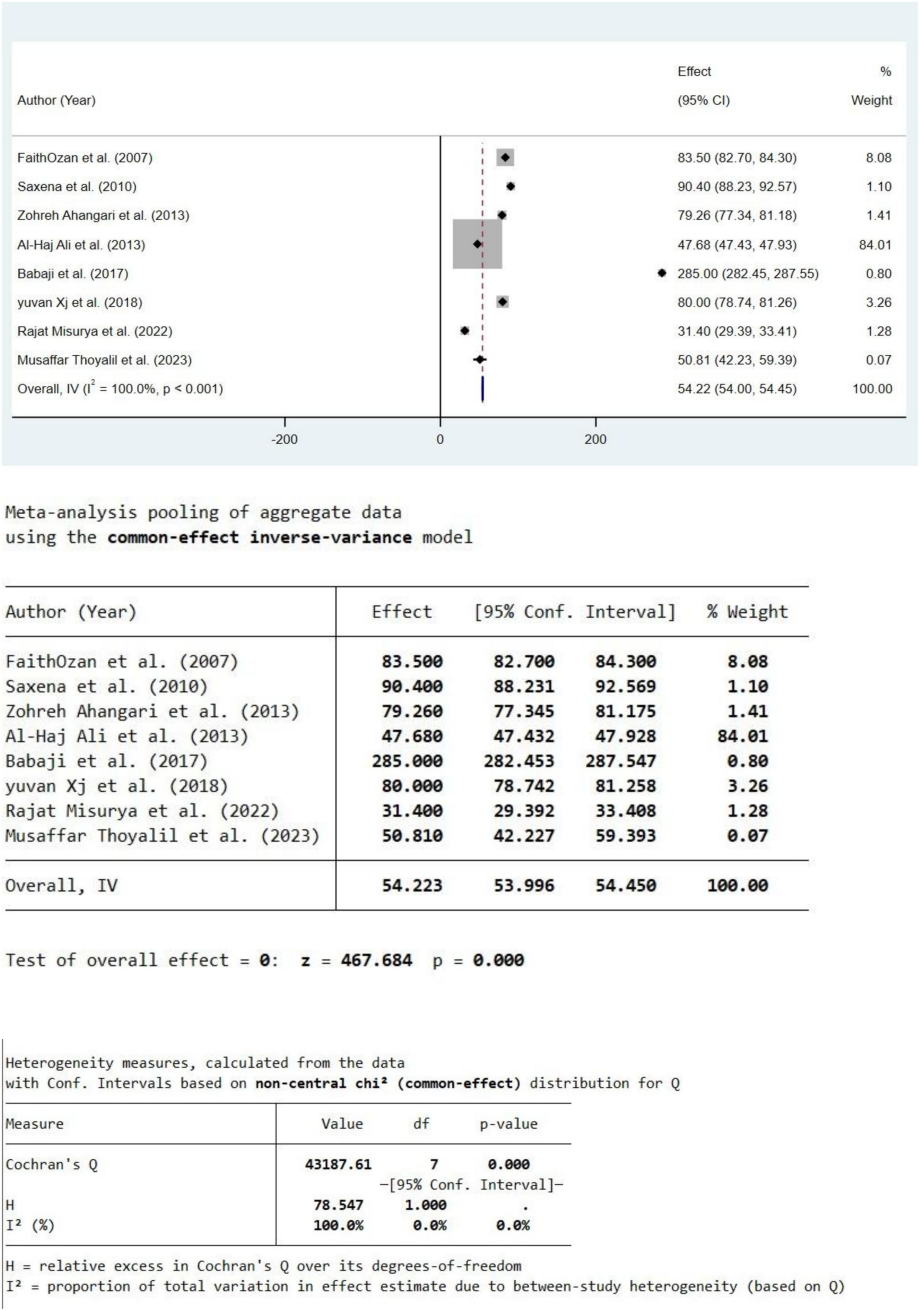
Forest plot of individual study effects and overall pooled effect size for 10% propolis.

The meta-analysis of 10% propolis demonstrates a statistically significant overall effect with a pooled estimate of 54.22 (95% confidence interval [CI]: 54.00 to 54.45) under a fixed-effect inverse-variance model, strongly rejecting the null hypothesis (Z = 467.684*Z* = 467.684, *p* < 0.001*p* < 0.001). This indicates a consistent biological activity associated with 10% propolis across the included studies. However, this pooled result is heavily influenced by one study (5), which accounts for 84% of the total weight, thereby dominating the meta-analytic estimate (Figure 2).

Despite the significant pooled effect, the heterogeneity among studies is extreme and statistically significant, as evidenced by Cochran’s Q = 43187.61*Q* = 43187.61 (*p* < 0.001*p* < 0.001) and I2 = 100%*I*2 = 100%, indicating that all observed variability is due to true differences between studies rather than sampling error. Individual study effect sizes vary widely, ranging from 31.40 to 285.00, with minimal overlap in their confidence intervals; this marked heterogeneity reflects substantial variation in study designs, populations, propolis sources, extraction methods, or outcome measurements.

The high heterogeneity raises concerns about the appropriateness of the fixed-effect model because it assumes a common true effect across studies. The observed variability suggests that a random-effects model, which accounts for between-study variance, would be more suitable for reliable inference. The extreme differences in effect sizes and study weights imply potential methodological or clinical heterogeneity, such as variations in chemical composition linked to geographical origin and extraction methods, both well recognized in propolis research.

The current systematic review identified significant variability in the methodologies used to assess PDL cell viability. Some studies utilized combination media, adding further complexity to the comparability of results. Due to substantial variations in the concentrations of propolis and the control groups used across the included studies, a meta-analysis of all data was not feasible. However, a forest plot was generated for studies that exclusively used 10% propolis, as this was the most consistently reported concentration. For other concentrations, the data were too heterogeneous to allow meaningful pooling. Instead, a qualitative synthesis was conducted to summarize the findings and provide a comprehensive overview of the available evidence.

## Discussion

Dental avulsion, is recognized as one of the most severe forms of traumatic dental injury, presenting significant clinical challenges in dental practice (20). A critical issue in managing avulsion is the loss of periodontal ligament (PDL) cell viability, which is a vital factor influencing the long-term success of tooth replantation. Immediate replantation is considered the gold standard for managing avulsed teeth as it helps sustain the viability of PDL cells, which are crucial for the reintegration and survival of the replanted tooth. However, in scenarios where immediate replantation is unachievable, placing the avulsed tooth in an appropriate storage medium can play a pivotal role in preserving the viability of PDL cells until replantation is feasible (21). One of the persistent challenges in dental traumatology lies in identifying the most effective storage medium for avulsed teeth. Over the years, researchers have investigated this issue using diverse experimental setups, including animal models such as dogs, monkeys, cats, rats, and mice, as well as human models utilizing healthy PDLs from extracted teeth (22). Among the various storage media tested, options like coconut water, Hank’s Balanced Salt Solution (HBSS), milk, pomegranate extract and others have been commonly studied for their efficacy in maintaining PDL cell viability. Interestingly, natural products have demonstrated superior results compared to synthetic alternatives, marking them as promising candidates in this domain (23).

A comprehensive Network Meta-analysis was conducted to evaluate the efficacy of 10 different storage media and identify the optimal medium for clinical use in preserving avulsed teeth before replantation. Based on ranking probabilities, propolis emerged as the most effective storage medium, outperforming all other commonly tested alternatives in the review (24). Despite its promising potential, no prior systematic review has focused exclusively on the role of propolis in different concentrations as a good storage medium for exarticulation. To bridge this research gap, the present systematic review was meticulously designed with stringent criteria to gather and analyze studies involving PDL cell cultures. This approach ensures a robust comparison of findings from the included studies, offering meaningful insights.

A total of 11 studies were identified through a detailed search strategy. These studies evaluated the effects of different storage media on PDL cell cultures at various time intervals. Each study was critically assessed using the PICO framework, which included the following components: Population (P): Human PDL cells isolated from freshly extracted teeth, Intervention (I): Propolis, Comparison (C): Control groups and other storage media (e.g., HBSS, milk, coconut water), Outcome (O): PDL cell viability, and Study Design (S): *In vitro* studies.

Propolis, a natural resinous substance produced by honeybees, has gained attention for its remarkable biological properties, including antioxidant, anti-inflammatory, and antimicrobial activities. In the field of dentistry, it has been explored for diverse applications, such as caries prevention in response to white spot lesion and also serving as an intracanal medicament during endodontic obturations, and acting as a storage medium for avulsed teeth in terms of traumatic injury. Its efficacy in maintaining the viability of Periodontal ligament cells can be attributed to its rich composition of biologically active compounds, including amino acids, vitamins, minerals, phenolics, and flavonoids, which collectively contribute to its therapeutic potential (25, 26).

Among the studies analyzed in this review, Brazilian propolis was the most frequently investigated type. Most of the propolis used in these studies was derived from plants, trees, and other natural sources associated with the honeybee species *Apis mellifera* L. One study also explored Jordanian propolis, which originates from *Boswellia serrata* and is linked to *Apis cerana*, a species of Asian honeybee (27, 28).

The methodologies employed to assess PDL cell viability varied across the studies, complicating direct comparisons. The most widely used method was the Trypan blue exclusion or staining test, which involves a 0.4% Trypan blue solution. This dye stains non-viable cells, allowing viable cells to be distinguished by their exclusion of the dye. While the Trypan blue test is regarded as a reliable method for determining cell viability, it has certain limitations. For instance, the dye’s cytotoxic nature and its potential to stain the background may introduce inaccuracies in cell counting (29, 30). Another technique employed was the MTT assay (3-[4,5-dimethylthiazol-2-yl]-2,5-diphenyl tetrazolium bromide), a colorimetric test that evaluates cellular metabolic activity. This method is advantageous due to its rapid results, objectivity, and simplicity, providing immediate and reliable identification of viable cells. Furthermore, its results are often comparable to histological examinations of PDL cells *in vitro* (31, 32). However, the MTT assay was used in only one of the studies included in this review (5).

The findings from this systematic review underscore the potential of propolis as an excellent storage medium for avulsed teeth, particularly in maintaining PDL (periodontal ligament) cell viability at concentrations. Considering that avulsed teeth are often exposed to environmental contamination, the antibacterial properties of propolis warrant further investigation (33). All studies in this review utilized propolis dissolved in a 0.4% ethanolic solution, a factor that may have influenced the results due to the pharmacological effects of ethanol. However, none of the studies specifically evaluated the antibacterial efficacy of this solution.

The risk of bias (ROB) for the included studies was assessed using the QUIN tool, and all studies demonstrated a low risk of bias. This robust assessment enhances the credibility of the review, as previous *in vitro* systematic reviews have rarely incorporated risk of bias evaluations. Nonetheless, the study had certain limitations. For example, the search was restricted to full-text articles available in English, which may have excluded relevant research published in other languages. Additionally, as the majority of the studies were conducted in India and Saudi Arabia, caution must be exercised when generalizing these findings to other regions.

Our literature review findings indicate that 10% propolis is highly effective in maintaining the viability of periodontal ligament (PDL) cells, particularly within a 30 min extra-oral dry time, showing a significant percentage of viable cells. Additionally, propolis demonstrates the ability to sustain cell viability for extended durations. However, as these results are derived from *in vitro* studies, it is essential to validate these findings through *in vivo* research.

Clinicians are encouraged to consider these promising results while remaining cautious about their direct application until further validation is available. Policymakers and researchers should prioritize developing clinical protocols that incorporate 10% propolis as a storage medium if future studies corroborate these findings.

Furthermore, training programs for healthcare providers should highlight the potential of propolis as a viable option for managing avulsed teeth, emphasizing its accessibility and efficacy.

“The results of this systematic review have important implications for clinical practice, policy development, and future research. Clinicians should rely on evidence-based media for PDL cell preservation, while policymakers should focus on standardizing protocols and improving access to propolis as an innovative storage media. Future research must address the methodological heterogeneity in current studies, explore the long-term outcomes of propolis, and conduct robust *in vivo* studies to establish its practical utility in real-world clinical settings.

## Conclusion

According to the available evidence, 10% Propolis proves to be more effective in preserving the viability of PDL cells compared to other storage media, making it a superior alternative. This systematic review also highlights potential areas for further research and recommends conducting in vivo studies with different time intervals to validate the clinical impact of Propolis on PDL cells.

## Data Availability

The original contributions presented in the study are included in the article/supplementary material, further inquiries can be directed to the corresponding author.

## References

[1] Lakhera H Mantri VR Palekar A Raut AW. Intraradicular rehabilitation of fractured maxillary anterior teeth with an open apex: a case report. J Indian Acad Dent Spec Res. (2016) 3:17–21. doi: 10.4103/2229-3019.192465

[2] Petrovic B Marković D Peric T Blagojevic D. Factors related to treatment and outcomes of avulsed teeth. Dent Traumatol. (2010) 26:52–9. doi: 10.1111/j.1600-9657.2009.00836.x, PMID: 19919541

[3] Pujita C Nuvvula S Shilpa G Nirmala S Yamini V. Informative promotional outcome on school teachers' knowledge about emergency management of dental trauma. J Conserv Dent. (2013) 16:21–7. doi: 10.4103/0972-0707.105293, PMID: 23349571 PMC3548340

[4] Patel D Mistry H Patel C Shah A Parikh V Mistry S. Comparative evaluation of periodontal ligament cell viability of permanent teeth in five different storage media followed by simulated avulsion injury: an in vitro study. Int J Health Sci. (2022) 6:1699–709. doi: 10.53730/ijhs.v6nS5.9022

[5] Ahangari Z Alborzi S Yadegari Z Dehghani F Ahangari L Naseri M. The effect of propolis as a biological storage media on periodontal ligament cell survival in an avulsed tooth: an *in vitro* study. Cell J. (2013) 15:244–9. 24027666 PMC3769607

[6] Misurya R Sharma S Syed Ismail PM Gupta N Rajan R Kaur R . An in vitro evaluation of efficacy of ViaSpan, *Aloe vera*, Gatorade solution, and propolis storage media for maintaining the periodontal ligament cell viability. Ann Afr Med. (2022) 21:34–8. doi: 10.4103/aam.aam_71_20, PMID: 35313402 PMC9020624

[7] Booth A Clarke M Dooley G Ghersi D Moher D Petticrew M . PROSPERO at one year: an evaluation of its utility. Syst. Rev. (2011) 2:4. doi: 10.1186/2046-4053-2-4 PMC3563608 23320413

[8] Shea BJ Reeves BC Wells G Thuku M Hamel C Moran J . PROSPERO at one year: an evaluation of its utility. BMJ. (2013) 358:Article j4008. doi: 10.1136/bmj.j4008

[9] Behera M Meher D. Zotero: an overview of open source citation management tool for researchers. Indian J Inf Libr Soc. (2022) 35:74–82.

[10] Sheth VH Shah NP Jain R Bhanushali N Bhatnagar V. Development and validation of a risk-of-bias tool for assessing in vitro studies conducted in dentistry: the QUIN. J Prosthet Dent. (2024) 131:1038–42. doi: 10.1016/j.prosdent.2022.05.019, PMID: 35752496

[11] Durán Ojeda G Bresser RA Wendler M Gresnigt MMM. Ceramic partial laminate veneers in anterior teeth: a literature review. J Prosthodont Res. (2024) 68:246–54. doi: 10.2186/jpr.JPR_D_23_00090, PMID: 37648480

[12] Martin M Pileggi R. A quantitative analysis of Propolis: a promising new storage media following avulsion. Dent Traumatol. (2004) 20:85–9. doi: 10.1111/j.1600-4469.2004.00233.x, PMID: 15025690

[13] Ozan F Polat ZA Er K Ozan U Değer O. Effect of propolis on survival of periodontal ligament cells: new storage media for avulsed teeth. J Endodont. (2007) 33:570–3. doi: 10.1016/j.joen.2006.12.021, PMID: 17437874

[14] Gopikrishna V Baweja PS Venkateshbabu N Thomas T Kandaswamy D. Comparison of coconut water, propolis, HBSS, and milk on PDL cell survival [retracted in: J Endod. 2014 Feb;40(2):290. doi: 10.1016/j.joen.2008.01.018]. J Endodont. (2008) 34:587–9. doi: 10.1016/j.joen.2008.01.018 18436040

[15] Saxena P Pant VA Wadhwani KK Kashyap MP. Potential of the propolis as storage medium to preserve the viability of cultured human periodontal ligament cells: an in vitro study. Dent Traumatol. (2011) 27:102–8. doi: 10.1111/j.1600-9657.2011.00974.x, PMID: 21385312

[16] Al-Haj Ali SN Al-Jundi S Mhaidat N. Comparison of coconut water and Jordanian Propolis on survival of bench-dried periodontal ligament cells: an in vitro cell culture study. Int J Clin Pediatr Dent. (2013) 6:161–5. doi: 10.5005/jp-journals-10005-1211, PMID: 25206215 PMC4086597

[17] Babaji P Melkundi M Devanna R S SB Chaurasia VR V GP. *In vitro* comparative evaluation of different storage media (hank's balanced salt solution, propolis, *Aloe vera*, and pomegranate juice) for preservation of avulsed tooth. Eur J Dent. (2017) 11:71–5. doi: 10.4103/ejd.ejd_101_16, PMID: 28435369 PMC5379839

[18] Yuan X Wang Y Shi B Zhao Y. Effect of propolis on preserving human periodontal ligament cells and regulating pro-inflammatory cytokines. Dent Traumatol. 34:245–53. doi: 10.1111/edt.12411 29806101

[19] Shingare P Chaugule V. Comparative evaluation of behaviors of three naturally occurring products, namely propolis, milk, and egg albumin when used as storage media in extracted teeth for orthodontic purpose. Arch Trauma Res. (2020) 9:129–34. doi: 10.4103/atr.atr_16_20

[20] Thoyalil M Belchada D Bidya Devi K Belchada DK Vasantha Ravi R Madathikandy Uchummal M . The comparative analysis of the effectiveness of four different storage media (Placentrex, Propolis 10%, pomegranate juice 5%, and Hank's balanced salt solution) in preserving the viability of periodontal ligament cells: an *in vitro* study. Cureus. (2023) 15:e42996. doi: 10.7759/cureus.42996, PMID: 37671229 PMC10476884

[21] Souza BDM Garcia LFR Bortoluzzi EA Felippe WT Felippe MCS. Effects of several storage media on viability and proliferation capacity of periodontal ligament cells. Eur Arch Paediatr Dent. (2020) 21:53–9. doi: 10.1007/s40368-019-00450-8, PMID: 31104259

[22] Osmanovic A Halilovic S Kurtovic-Kozaric A Hadziabdic N. Evaluation of periodontal ligament cell viability in different storage media based on human PDL cell culture experiments-a systematic review. Dent Traumatol. (2018) 34:384–93. doi: 10.1111/edt.12437, PMID: 30193009

[23] Chandakavathe BN Kulkarni R Dhadde SB. Formulation and assessment of in vitro antimicrobial activity of herbal toothpaste. Proc. Natl. Acad. Sci. India, Sect. B Biol. Sci. (2022) 93:317–23. doi: 10.1007/s40011-022-01424-5

[24] Zhang N Cheng Y Li F Kang Q. Network Meta-analysis of 10 storage mediums for preserving avulsed teeth. Front Med. (2021) 8:749278. doi: 10.3389/fmed.2021.749278, PMID: 34708058 PMC8542672

[25] Longo DL Fumes AC Küchler EC Paula-Silva FWG Nelson-Filho P Silva LAB. Efficiency of different storage media for avulsed teeth in animal models: a systematic review. Dent Traumatol. (2018) 34:12–9. doi: 10.1111/edt.12365, PMID: 28853235

[26] Adnan S Lone MM Khan FR Hussain SM Nagi SE. Which is the most recommended medium for the storage and transport of avulsed teeth? A systematic review. Dent Traumatol. (2018) 34:59–70. doi: 10.1111/edt.12382, PMID: 29292570

[27] Esh S Chatterjee AN De B. Propolis and its implications in dentistry: a review. Int J Res Rev. (2021) 8:311–7. doi: 10.52403/ijrr.20211238

[28] Kaur N Srivastava N Rana V Kaushik N Pruthi T. Storage medium for avulsed teeth: a literature review. Int J Adv Res. (2021) 9:874–86. doi: 10.21474/IJAR01/13340

[29] Sanghavi T Shah N Parekh V Singbal K. Evaluation and comparison of efficacy of three different storage media, coconut water, propolis, and oral rehydration solution, in maintaining the viability of periodontal ligament cells. J Conserv Dent. (2013) 16:71–4. doi: 10.4103/0972-0707.105303, PMID: 23349581 PMC3548351

[30] Saini D Gadicherla P Chandra P Anandakrishna L. Coconut milk and probiotic milk as storage media to maintain periodontal ligament cell viability: an in vitro study. Dent Traumatol. (2017) 33:160–4. doi: 10.1111/edt.12310, PMID: 27943593

[31] Mosmann T. Rapid colorimetric assay for cellular growth and survival: application to proliferation and cytotoxicity assays. J Immunol Methods. (1983) 65:55–63. doi: 10.1016/0022-1759(83)90303-4, PMID: 6606682

[32] Kim H-K Kim E-S Choi I-B Kim J Lee S-J. The verification of the MTT assay on the viability of periodontal ligamental cells in rat molars through the histologic examination. J Korean Acad Conserv Dent. (2003) 28:385–91. doi: 10.5395/JKACD.2003.28.5.385

[33] Resende KKM Faria GP Longo DL Martins LJO Costa CRR. In vitro evaluation of plants as storage media for avulsed teeth: a systematic review. Dent Traumatol. (2020) 36:3–18. doi: 10.1111/edt.12501, PMID: 31328384

